# Supplementary material for: The role of objectively recorded smartphone usage and personality traits in sleep quality
Source: PeerJ Comput Sci. 2023 Mar 27;9:e1261. doi: 10.7717/peerj-cs.1261 (PMC10280441; doi:10.7717/peerj-cs.1261)
Supplement: Supplemental Information 1 [file peerj-cs-09-1261-s001.zip › Variable explanation.docx]

**Variables Explanation**

| **Variable** | **Explanation** |
| --- | --- |
| Age | Age range of the participant,  15-25,  25-34 and so on. |
| **Age_Coded_Bin** | A variable that represents two categories, i.e.,  Emerging Adults (15-24)  Adults (25-64). |
| Gender | Male or Female |
| **Gender_Coded** | Codes for gender, i.e.,  1= Male  2 = Female |
| Country | Represents participants country. |
| **Extraversion** | Big Five Inventory‐10 (BFI‐10) (Rammstedt and John, 2007). This self-report measure includes 10 items, loading on five factors, assessing five dimensions of personality: extraversion, agreeableness, conscientiousness, neuroticism, and openness to new experience. |
| **Agreeableness** |  |
| **Conscientiousness** |  |
| **Neuroticism** |  |
| **Openness** |  |
| **UsageOfAppsAvg** | In this paper, we called this variable **UsageOfAppsAvg**. This is an independent continuous variable. We first calculated the smartphone usage of each participant for one week and then calculate the daily average usage in terms of minutes. UsageOfAppsAvg represents the daily average usage of smartphone apps. |
| **SDur** | This is the average sleeping time of a participant. In paper, we called it SDur. SDur is a continuous variable representing the daily average duration of the sleeping hours. We estimated the sleep duration of each night from the smartphone usage data and identified the non-usage period in minutes during the sleep-wake cycle. |
| **SDurQ** | Based on the literature (Hirshkowitz *et al.*, 2015), we create SDurQ categorical variable from SDur. SDurQ represents the sleep duration quality and consists of **three** categories, i.e., poor e-sleep (if SDur is less than 7 h), good e-sleep (if SDur is between 7 h and 9 h), and over e-sleep (if SDur is greater than 9 h). |
| **SDist** | SDist is a continuous variable representing the daily average duration in minutes of the distraction caused by smartphones during sleeping hours. |
| **SDistC** | In this paper, this variable is called **SDistC**. Distraction happens when attention is given to the distracting object for more than eight seconds (Kelly Howard, 2019). Thus, based on SDist (**DistractedSessionsAverage**) (, we created SDistC categorical variable. SDistC has two categories, i.e., not-distracted (if the distraction duration during sleeping hours is less than 0.13 minutes, i.e., SDist < 8 seconds), and distracted (if the distraction duration is equal or greater than 0.13 minutes, i.e., SDist ≥ 8 seconds). |
| SleepingTimeAverageWeekly | This is the actual sleeping time in terms of time stamp and were used to calculate SleepingTimeWeeklyCategory and STime. |
| SleepingTimeWeeklyCategory | Sleeping time categories, i.e., Poor Sleep Time, Delayed Sleep Time, Regular Sleep Time, and Early Sleep Time. |
| **STime** | In this paper we call this variable STime. Based on the literature (Yan *et al.*, 2021), we create STime categorical variable with four categories, i.e., early sleep (< 10:00 PM), regular sleep (10:01 PM to 11:00 PM), delayed sleep (11:01 PM to 12:00 AM), and poor sleep (> 12:01 AM). |
| WakeupTimeWeeklyAverage | This is the daily average wakeup time of the participants. |
| WakeupTimeWeeklyCategory | we created this variable with four categories, i.e., early wakeup (< 07:00 AM), regular wakeup (07:01 AM to 08:00 AM), delayed wakeup (08:01 AM to 09:00 AM), and poor wakeup (> 09:01 AM). |
| **WTime** | In this paper, we call it **WTime**.  WTime is a categorical variable code based on WakeupTimeWeeklyCategory.  WTime is a categorical variable representing the average wakeup time of the participants. Based on the literature (Yan *et al.*, 2021), we created a WTime variable with four categories, i.e., early wakeup (< 07:00 AM), regular wakeup (07:01 AM to 08:00 AM), delayed wakeup (08:01 AM to 09:00 AM), and poor wakeup (> 09:01 AM). |
